# Completeness of tuberculosis case notifications in Germany in 2013–2017: first results of an inventory study

**DOI:** 10.1186/s12879-020-05467-9

**Published:** 2020-10-17

**Authors:** Teresa Domaszewska, Basel Karo, Ute Preuss, Christian Kollan, Annicka Reuss, Hans-Peter Blank, Bonita Brodhun, Barbara Hauer, Doris Altmann, Lena Fiebig, Walter Haas, Nita Perumal

**Affiliations:** 1 Department for Infectious Disease Epidemiology, Robert Koch Institute, Berlin, Germany; 2 Robert Koch Institute, Centre for International Health Protection (ZIG), Berlin, Germany; 3 Current affiliation: APOPO, Sokoine University of Agriculture, Morogoro, Tanzania

**Keywords:** Tuberculosis, Reporting completeness, Inventory study, Surveillance, Underreporting

## Abstract

**Background:**

Evaluating the completeness of tuberculosis (TB) notification data is important for monitoring of TB surveillance systems. We conducted an inventory study to calculate TB underreporting in Germany in 2013–2017.

**Methods:**

Acquisition of two pseudonymized case-based data sources (national TB notification data and antibiotic resistance surveillance data) was followed by two-source Capture-recapture (CRC) analysis, as case-based data from a third source was unavailable. Aggregated data on consumption of a key anti-TB drug (pyrazinamide [PZA]) was compared to an estimated need for PZA based on TB notification data to obtain an independent underreporting estimation. Additionally, notified TB incidence was compared to TB rate in an aggregated health insurance fund dataset.

**Results:**

CRC and PZA-based approaches indicated that between 93 and 97% (CRC) and between 91 and 95% (PZA) of estimated cases were captured in the national TB notification data in the years 2013–2017. Insurance fund dataset did not indicate TB underreporting on the national level in 2017.

**Conclusions:**

Our results suggest that more than 90% of estimated TB cases are captured within the German TB surveillance system, and accordingly the TB notification rate is likely a good proxy of the diagnosed TB incidence rate. An increase in underreporting and discrepancies however should be further investigated.

## Background

Tuberculosis (TB) is one of the biggest public health infectious disease threats globally, with an estimated 10 million people who fell sick with TB and 1.6 million people who died of TB in 2017 [1]. To achieve the goals of the End TB strategy of the World Health Organization (WHO) [2] it is crucial to diagnose people with TB early and treat them adequately. Mandatory case-based reporting to a national surveillance system allows systematic and regular monitoring and treatment follow-up of TB patients. Yet, it is estimated that, in 2017, approximately 35% of TB patients worldwide were either not diagnosed and/or not reported to surveillance systems [1]. Evaluation of the reliability of TB surveillance is important for development of national strategies, tracking and reporting progress in control efforts, and guiding policy decisions. Surveillance data should reflect the real number of disease cases, as the consequence of underreporting is uncertainty in the key indicators for TB, such as the incidence and treatment outcome [3, 4].

In Germany, the Robert Koch Institute (RKI), as the national public health institute, is responsible for conducting nationwide infectious disease surveillance. Since TB is a notifiable disease [5], pseudonymized case-based data on TB patients is reported to the national surveillance database of the RKI (SurvNet@rki [6]) by the local district health authorities via state health departments. The TB notification rate in Germany was on the rise between 2013 and 2016 with the largest increase in 2015 (TB incidence, unit: persons/100.000 population per year; in 2013: 5.4; 2014: 5.6; 2015: 7.1; 2016: 7.2; number of notified TB patients in 2013: 4345; 2014: 4529; 2015: 5837; 2016: 5926). In 2017, 5495 TB patients were notified to the RKI, corresponding to reported incidence of 6.6 per 100,000 population. Using standard adjustment of the notification data, the WHO estimated the percentage of TB underreporting in Germany to be 11% for 2017 [1]. However, assessing reporting completeness of the electronic surveillance systems is best done through inventory studies [7], which reveal data-driven and, hence, more certain underreporting estimates that are tailored to each country’s unique health care system and TB epidemiology.

An inventory study is a methodology used to investigate the level of underreporting of cases of a disease based on comparison of the records in independent disease registers [4]. It includes acquisition of case-based datasets, de-duplication of each data source, and record-linkage (RL). RL can either be followed by capture-recapture (CRC) analysis or observed underreporting calculation [4].

In recent years the gap between reported and real TB incidence has been investigated through the use of inventory studies in China [8], Egypt [9], Indonesia, Iraq [10], Taiwan [11], Yemen [12] and nine European countries [13–17]. In Greece the reporting completeness was calculated from the recorded anti-TB medication consumption [18].

To date, no inventory study has been conducted in Germany to estimate the underreporting of TB patients and the TB underreporting estimated by WHO has not been validated using the existing case-based data sources. Here, we aim to conduct an inventory study for diagnosed TB in Germany by acquiring available case-based datasets and estimating TB underreporting in Germany for the years 2013–2017 in order to estimate the completeness of the German TB notifications.

## Methods

The study design is an inventory study using CRC methodology, which stems from ecology and allows modeling of the total population size [19]. In the field of epidemiology, this method is implemented to estimate the completeness of disease registers [20]. The assumptions of CRC are: closed population structure, independence of data sources, perfect matching across data sources, and the same probability of being included in different data sources. Since CRC studies in epidemiology rarely meet all of these assumptions, it is recommended to use at minimum three data sources in order to counteract the effect of the violation of assumptions on the study results [4].

In a different approach, the number of disease cases can be calculated based on recorded utilization of disease-specific medication defined by anatomical-therapeutic-chemical (ATC) classification system controlled by WHO Collaborating Centre for Drug Statistics Methodology [21] in a given time and study area [15]. Data on utilization of a particular medication can include medication sales, number of filled prescriptions, or number of written prescriptions. Defined daily dose (DDD) and average treatment time define the amount of the medication needed to treat a patient [22]. Out of the four first-line drugs used in the treatment of TB, Pyrazinamide (PZA) is the only medication used exclusively to treat TB and no other medical conditions and is used primarily in the intense treatment phase. Hence, it is also possible to use the total amount of PZA utilized over a particular time period in a region or country as a proxy for the total number of the treated TB patients, after adjusting for important factors that affect the utilization, such as resistances.

**Fig. 1:**
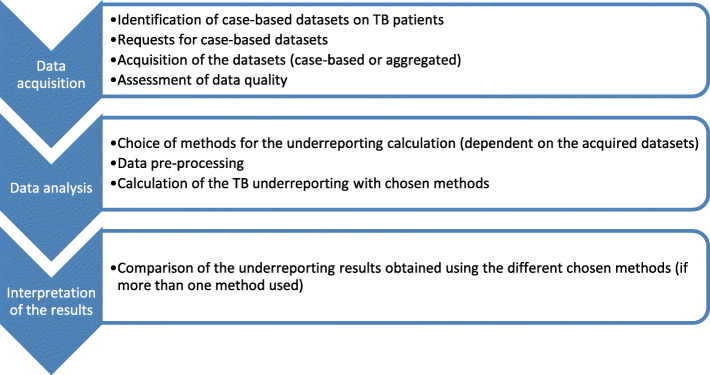
Design of the inventory study of TB registers in Germany

Figure 1 summarizes the design and steps of the TB inventory study in Germany, as described in detail below.

### Data acquisition

Only institutions from which the datasets were acquired and used in the study are named in this manuscript. The remaining ones are categorized as particular institution type (e.g. “statutory health insurance company”).

#### Identification of case-based datasets on TB patients and requests for case-based datasets

A list of 23 institutions that collect case-based data including data on TB patients in Germany was created. Institutions with data sharing policies that prevented the acquisition of case-based data within the project timeline and/or institutions lacking recent data were excluded from the list. Inquiries for anonymized, case-based datasets were sent to two statutory health insurance (SHI) companies, a health insurance fund, two statistical agencies, a research institute for medical care in Germany, and, internally to a unit of RKI (Supplementary Table 1). One aggregated dataset with PZA pharmacy data was already available at RKI.

#### Acquisition of the datasets (case-based or aggregated)

Out of the contacted institutions, only the RKI Unit for Healthcare-associated Infections, Surveillance of Antibiotic Resistance and Consumption accepted the inquiry to share national, case-based antibiotic resistance surveillance (ARS) data. Among the contacted external institutions, three agreed to send only aggregated data due to internal data-sharing policies, one rejected the data-sharing request due to lack of capacity, one required a fee and had a waiting time that exceeded the funding period of the study, and one stopped responding to e-mails or calls with no data being sent (Supplementary Table 1). By March 2019, the data collection was completed with the acquisition of two case-based pseudonymized datasets, both of which are held by the RKI: national TB notification data and ARS data. Five aggregated datasets from three external institutions were acquired. Data protection guidelines were strictly followed for all data received, whether case-based or aggregated. The TB notification data utilized in the study were collected in accordance with the German ‘Protection against Infection Act’ (‘Infektionsschutzgesetz’), which has very stringent data protection guidelines.

#### Assessment of data quality

Both case-based datasets contained variables that were necessary for matching the two datasets and were included into the further analysis. Out of the five aggregated datasets, three were excluded from the analysis due to unreliable data quality (e.g. ratio of female to male TB patients larger than 1:1, although the female to male ratio among TB patients has been consistently reported as being close to 1:2 [1, 23, 24]). Aggregated data from Betriebskrankenkassen Dachverband (BKK DV) – health insurance fund – and from Insight Health, a company which collects prescription data from pharmacies - were included into the further analysis.

#### Data sources

##### TB notification data

The TB patients in TB notification data are defined by the RKI case definition [25] and meet at least one of the two criteria: the responsible medical doctor recommended full course of anti-TB treatment, or evidence was found for the need of full antibiotic treatment of TB after the death of a person. The case definition corresponds to ICD-10 codes: A15-A19.9; B90.0-B90.9; U82.0 and U82.1 For the purposes of this study, a subset of the TB notification data (data status as of March 31st, 2019) spanning the years 2013–2017 and containing the following variables was extracted: birth month and year, sex, federal state, hospitalization status and dates, site of disease, date and month of death (if applicable), and resistance to any of the following anti-TB medications: PZA, isoniazid, rifampicin, ethambutol, streptomycin, ofloxacin, moxifloxacin, levofloxacin, amikacin, kanamycin, capreomycin, cycloserine, linezolid, p-aminosalicylic acid, prothionamide, or rifabutin.

##### Antibiotic resistance surveillance (ARS) data

ARS is a surveillance system developed by RKI in 2007 [26]. It contains routine case-based data on antimicrobial susceptibility testing from voluntarily participating German laboratories. In the years 2013–2017 21 laboratories providing diagnostic services for altogether 179 hospitals and 3262 out-patient practices Germany-wide participated continuously in the data collection on TB. For the purpose of this study, one isolate with microbiologically confirmed TB was counted as one patient. A subset of the ARS data spanning the years 2013–2017 and containing the following variables was extracted: birth month and year, sex, federal state, hospitalization status, and resistance to any of the anti-TB medications: PZA, isoniazid, rifampicin, ethambutol, streptomycin, ofloxacin, moxifloxacin, levofloxacin amikacin, kanamycin, capreomycin, cycloserine, linezolid, p-aminosalicylic acid, prothionamide, or rifabutin.

##### PZA pharmacy data

Insight Health collects monthly data on prescriptions filled by pharmacies in Germany, which are purchased by the RKI in aggregated form. The database captures 99.8% of all pharmacy data within the SHI market in Germany, which covered between 86.5 and 87.2% of the German population over the study period, but does not include medications supplied in hospital pharmacies or in prisons. For the purposes of this study, a subset of the aggregated PZA prescription data spanning the years 2013–2017 and containing the following variables was extracted: federal state, medication pharmacy central number, medication producer, number of pills in a package, medication strength, and total number of filled prescriptions.

##### Insurance data

BKK DV is a central organization of company health insurance funds in Germany. As of March 2019, it includes 76 health insurance companies with 9 million insured individuals, constituting 10.6% of German population. BKK DV provided aggregated data on sex and state for individuals registered with ICD-10 codes corresponding to TB: A15-A19.9; B90.0-B90.9; U82.0 and U82.1 who were insured through one of the 76 insurance companies and underwent in-patient hospital treatment for TB in 2017.

### Data analysis

#### Choice of methods for the underreporting calculation

**Fig. 2:**
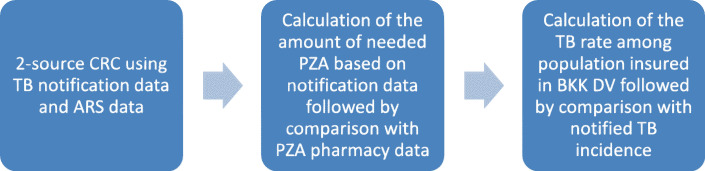
Methods used to calculate TB reporting completeness in Germany based on different collected datasets

We conducted a two-source CRC, since only two case-based datasets were available. In order to address possible impact of violation of the CRC assumptions on the study results, a novel, “double-pronged” approach was developed to validate the CRC results using independent aggregated data (Fig. 2). The PZA pharmacy data was utilized to independently derive the total number of active TB patients in each of the years between 2013 and 2017. The expected amount of utilized PZA was derived from TB notification data and compared to the actual amount of PZA utilized according to PZA pharmacy data to calculate TB reporting completeness. This way, the underreporting estimates resulting from CRC analysis could be triangulated using independently calculated TB underreporting based on the PZA pharmacy dataset. Additionally, the BKK DV insurance data was utilized to test whether the TB rate among the population insured by BKK DV in 2017 indicated TB underreporting in the 2017 TB notification data.

#### Data preprocessing

All analysis steps and calculations were conducted using R (version 3.5.1) [27]. Column and variable names were standardized between ARS and the TB notification data. Missing values for sex, hospitalization, and state variables were replaced with *NA* values. The ARS dataset was de-duplicated by retaining only entries with the earliest report date in cases where the same patient identifiers were recorded multiple times. All datasets were split by reporting years.

#### Calculation of the TB underreporting part I: record linkage, observed underreporting calculation and capture-recapture analysis

Probabilistic RL between TB notification data and ARS data for calculation of observed underreporting and CRC analysis was conducted in order to identify patients captured in both, the TB notification system and ARS database, using the R-package RecordLinkage [28] with birth month, birth year, federal state, hospitalization status, and sex as obligatory matching arguments in the first iteration of the matching. The matched patients were then excluded from the datasets and a second iteration of matching was performed for the remaining patients, excluding the obligatory matching of sex in order to include the patients with missing sex. In the same way, third and fourth iterations of matching were performed excluding the obligatory matching based on state and hospitalization variables, respectively. The pairs recorded in each iteration of the matching process were summarized to calculate overlap between the datasets.

The observed underreporting was calculated for each year as the sum of TB patients notified to the RKI for that year and the patients who were reported to ARS in that same year, minus the patients matched between the two databases.

CRC analysis of TB notification and ARS data was performed using R package Rcapture [23]. Closed population models were built for each of the reporting years from 2013 to 2017 using “closedp” function, with the vectors representing captures of patients in ARS and the TB notification data as arguments and default parameters. In each of the investigated years the model presenting minimal Akaike’s Information Criterion (AIC) and adjusting for lack of independence between the data sources was chosen to estimate the total number of TB patients in Germany (for additional details, see Supplement Method 1) and the 95% confidence intervals (95% CI).

#### Calculation of the TB underreporting part II: validation of CRC results via analysis of aggregated PZA data

Total recorded quantity of PZA from filled prescriptions in years 2013–2017 was calculated as sum of the prescribed standard units of the medication multiplied by their corresponding strength reported in PZA pharmacy data (Eq. 1):

1 $$ {A}_{PZA}={\sum}_{i=1}^nP\times {S}_{PZA} $$

Where:

A_PZA_ – total recorded amount of PZA from filled prescriptions;

P – number of standard units of PZA from filled prescriptions;

S_PZA_ – strength of PZA in one standard unit from filled prescriptions;

n – types of standard units of PZA from filled prescriptions in a given year.

Total amount of PZA required for treatment of all notified TB patients in Germany, 2013–2017, was estimated with two approaches. First, the amount of PZA needed to treat all notified TB patients was calculated according to a previously described WHO methodology on drug utilization research [24]. For this calculation, defined daily dose of PZA (DDD_PZA,_ 1750 mg) and an average treatment time of 60 days were used for all the patients notified in each of the years 2013–2017. However, since over 70% of TB patients in Germany underwent hospitalization in 2013–2017 according to the TB notification data (Supplement Table 1), and PZA treatment regimens vary depending on the patients’ age, weight, and antibiotic resistance profile, a second approach, where the amount of PZA needed to treat each notified patient was calculated based on (A) the estimated DDD_PZA_ adjusted for individual patient’s age, and (B) the treatment length adjusted for individual resistance profile, age, length of hospitalization, and death during treatment, was implemented. All treatment adjustments were based on the German national treatment guidelines for TB [29] and were confirmed with guidelines authors. The implemented adjustments are listed in the Table 1.

**Table 1:** Adjustments to the DDD_PZA_ and PZA treatment length based on notified patients’ characteristics

Adjustment to	Notified variable	Value of the notified variable	Adjustment
DDD_PZA_*	Age group	0–4	500 mg
		5–10	750 mg
		11–14	1250 mg
		> 14	1750 mg
Treatment length	Resistance against	Isoniazid + Ethambutol	10.5 months (average of the recommended 9 to 12 months)
		Isoniazid + Rifampicin**	18 months
		PZA	0 months
	Hospitalization	Hospital admission and discharge dates	Recommended PZA treatment time (60 days) minus number of days of hospitalization falling within PZA treatment time
		Hospitalization status = “yes”	Recommended PZA treatment time (60 days) minus average number of days of hospitalization*** falling within PZA treatment time
	Death during treatment	Date of death	Recommended PZA treatment time (60 days) minus number of days between date of death and expected treatment end time

*The adjustments to DDD_PZA_ were based on patient’s age since weight is not a notifiable variable. The dosage assumed for age groups was based on the recommendations of the medication producer Riemser Arzneimittel AG, Germany, which supplied the majority of PZA in Germany from 2013-2017 (according to the PZA pharmacy data)

**Multidrug-resistant TB (MDR-TB)

***Calculated based on hospitalized TB patients from the corresponding year

**Fig. 3:**
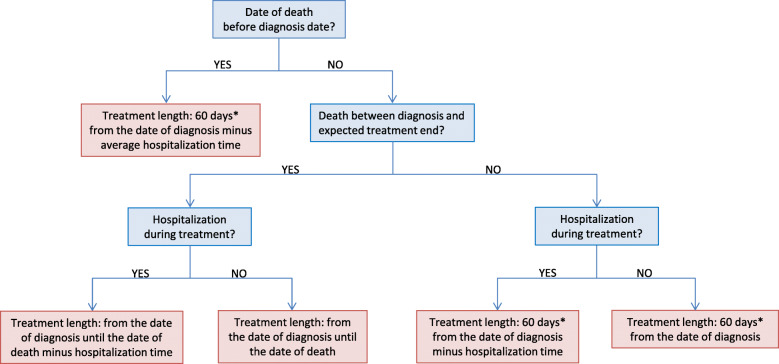
Algorithm used to estimate PZA treatment length based on TB notification data. The treatment time adjustments included the events of hospitalization within treatment time, death before the end of treatment, and resistances. In cases where TB notification data contained inconsistent information on date of death and/or date of diagnosis (implying that diagnosis took place after death), standard treatment length of 60 days with PZA and the average hospitalization time of all TB patients from the corresponding year were assumed. *For TB patients with resistance against PZA, the length of treatment with PZA was assumed to be 0 days. For patients with Isoniazid and Streptomycin resistance, the expected PZA treatment time was 315 days (10.5 months). For patients with MDR-TB, the PZA treatment length was considered 540 days (18 months)

The length of the treatment using PZA for each patient was calculated according to the algorithm presented in Fig. 3.

Adjusted DDD_PZA_ and treatment length were used to calculate PZA needed for every notified patient according to Eq. 2:

2 $$ { PZ A}_i={DDD}_{PZ{A}_i}\times {t}_i $$

Where:

*PZA*_*i*, *year*_ – PZA need for a given notified TB patient from a given year;

${DDD}_{PZA_{i, year}}$– DDD_PZA_ estimated for a given TB patient from the given year;

*t*_*i*, *year*_ – PZA treatment time for a given TB patient from the given year.

The calculated amount of PZA was summarized for patients notified in a given year as follows (Eq. 3):

3 $$ {PZA}_{year}={\sum}_{i=1}^n{PZA}_{i, year} $$

Where:

*PZA*_*year*_ – PZA needed for treatment of single notified TB patients in a given year;

*n* - number of TB patients in a given year.

The 95% CI was calculated using nonparametric bootstrapping with 10,000 bootstraps for data from each notification year with R-package boot [30, 31].

#### Comparison of TB notification data with insurance data of hospitalized TB patients

In 2017, 10.6% of German population was insured by BKK DV, with the proportion of those insured varying by federal state (Table 3). The population counts were based on the reports of Federal Statistical Office of Germany from March 2019 [32]. In order to compare the TB rates in the insurance data to the notified TB incidence for every state, the rate of population in every state insured by BKK DV was calculated, with exclusion of 4 out of 16 states (Thuringia, Bremen, Saarland and Mecklenburg-Western Pomerania, altogether constituting 6.6% of the German population), which was not shared by BKK DV due to internal data protection policies preventing sharing aggregated data in case of low case number. Then, patients that were notified as resulting from asylum seekers, refugee, prisoners and homeless people screening as well as patients diagnosed after death were excluded from the TB notification data, since they are not eligible to be insured by BKK DV and are covered under other insurance and/or medical payment schemes, as well as TB patients who were notified as not having been hospitalized (insurance data received consisted of hospital in-patient care data only). The patients diagnosed with TB after death would not have been registered as TB patients in BKK DV. Next, TB rates were calculated separately for every federal state, first using the insurance data and then using the TB notification data and the values obtained from the two data sources were compared.

## Results

### Probabilistic RL, observed underreporting and CRC analysis

**Fig. 4:**
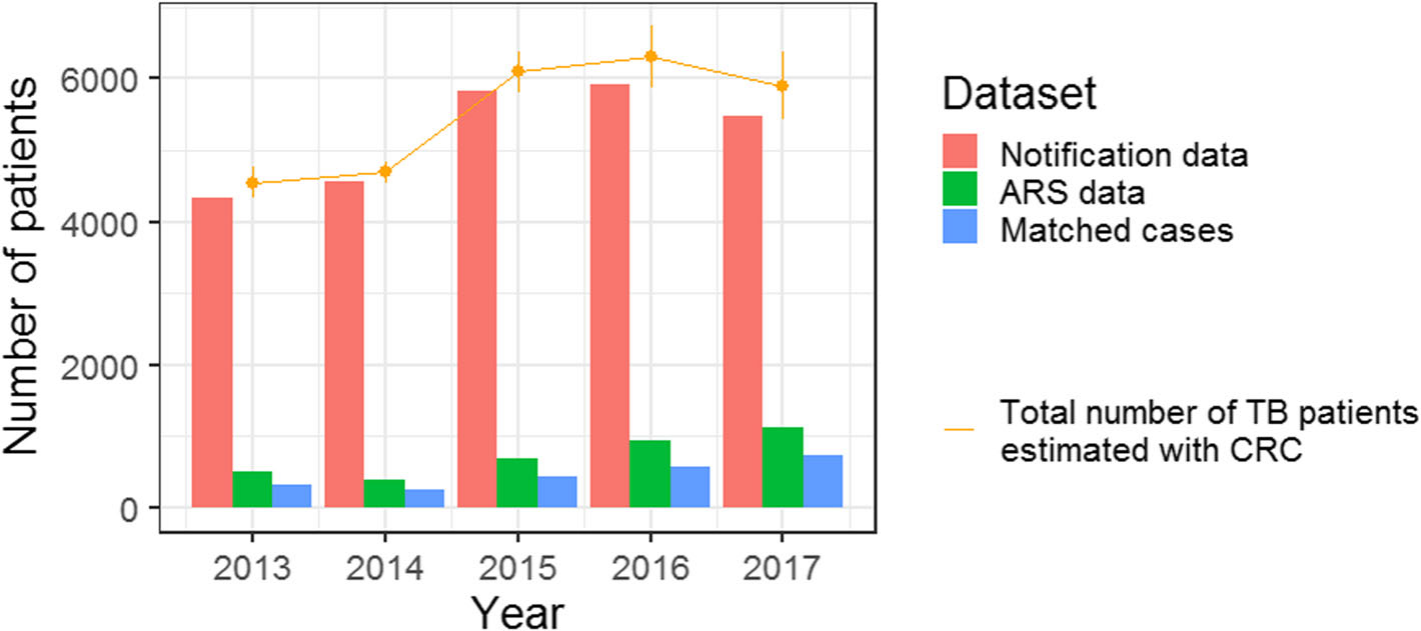
Underreporting of TB in Germany in years 2013–2017 estimated with CRC. The red columns represent number of notified TB patients. The green columns represent numbers of patients reported to ARS. The blue columns represent numbers of patients matched between the TB notification system and ARS. Yellow dots represent CRC estimation of the total number of TB patients in Germany in the years 2013–2017. Bars represent standard deviation

Probabilistic RL revealed high level of overlap between the TB notification and ARS registers (Table 2). The observed underreporting ranged from 2.9 to 6.5% and increased from 4.2 to 6.5% in 2013–2017. The underreporting estimated with CRC ranged from 3.0 to 7.0% and increased from 4.4 to 7.0% in 2013–2017 (Table 2, Fig. 4).

**Table 2:** The results of RL and CRC analysis for each of the years 2013–2017

Year	Number of notified patients	Number of ARS patients	Number of matched patients	Observed number of patients	Observed under-reporting	Population size (CRC)	Std. error (CRC)	Under-reporting (CRC)
2013	4345	503	310	4539	4.2%	4549; 95% CI: 4542-4554	3.3	4.4%; 95% CI: 4.3–4.6%
2014	4529	391	255	4666	2.9%	4672; 95% CI: 4542-4554	2.2	3.0%; 95% CI: 3.0–3.1
2015	5837	687	440	6084	4.0%	6095; 95% CI: 6088-6102	3.6	4.2%; 95% CI: 4.1–4.3
2016	5926	937	580	6283	5.7%	6306; 95% CI: 6295-6316	5.4	6.0%; 95% CI: 5.9–6.2
2017	5495	1124	741	5878	6.5%	5908; 95% CI: 5895-5919	6.2	7.0%; 95% CI: 6.8–7.2

*Std. error* standard error of the mean, *95% CI* 95% confidence interval

#### Estimation of TB underreporting in Germany based on PZA pharmacy data

**Fig. 5:**
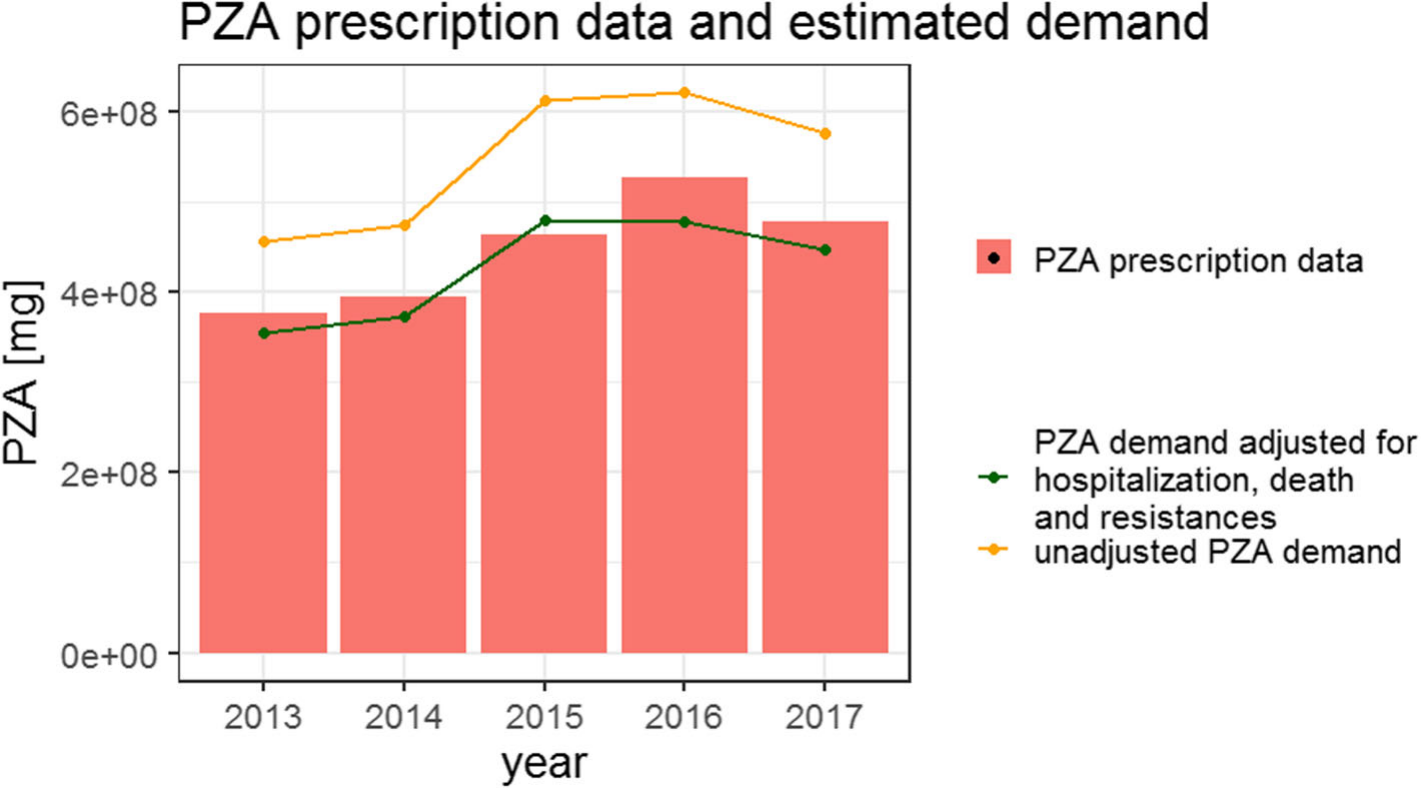
Underreporting of TB estimated based on prescribed PZA pharmacy data

The amount of PZA required to treat the notified TB patients and calculated according to the methodology on drug utilization research [33] was higher in comparison to the amount of PZA from filled PZA prescriptions in each of the years 2013–2017 (Fig. 5). However, after adjusting treatment length and DDD_PZA_ for the factors influencing the recommended duration of PZA treatment, the obtained amount of PZA needed to treat the notified patients in 2013–2017 was lower (Fig. 5). The difference between the amount of PZA sold by pharmacies and PZA demand adjusted for the listed factors demonstrated underreporting of TB in the years 2013, 2014, 2016 and 2017 (Table 3). In 2015, the estimated demand was still higher than the amount of PZA sold in pharmacies (Table 3).

**Table 3:** The results of PZA pharmacy data analysis for the years 2013–2017

Year	Number of notified patients	Average number of days of hospitalization per TB patient	Amount of PZA in PZA pharmacy data in 10^8^ mg	PZA needed for treatment of all patients reported in the notification year in 10^8^ mg	Underreporting
2013	4345	7.8	3.78	3.55; 95% CI: 3.44–3.65	6.0%; 95% CI: 3.4–7.8%
2014	4529	6.8	3.95	3.74; 95% CI: 3.64–3.83	5.3%; 95% CI: 3.0–7.8%
2015	5837	6.1	4.64	4.81; 95% CI: 4.68–4.91	− 3.6%; 95% CI: 1.0–5.7%
2016	5926	6.1	5.29	4.79; 95% CI: 4.66–4.89	9.4%; 95% CI: 7.5–11.8%
2017	5495	5.5	4.79	4.48; 95% CI: 4.32–4.56	6.6%; 95% CI: 4.8–9.8%

#### Comparisons of TB notification data with aggregated insurance data

We compared the TB notification rate with the TB rate the insurance data (Table 4). In all the states as well as for the whole country, rate of TB was lower in insurance data than in the TB notification data. Therefore, no underreporting on a country-level was inclined by aggregated insurance data.

**Table 4:** Comparison of TB notification data from 2017 with insurance data

State	Number of people insured in BKK DV	Number of TB patients in insurance data	Population	Number of notified TB patients	Adjusted^a^ number of notified patients	Population rate insured in BKK DV	TB rate^b^, insurance data	Adjusted^a^ TB notification rate^b^	TB notification rate^b^/TB rate^b^ insurance data
Schleswig-Holstein	203,013	8	2,889,821	137	118	0.07	3.94	4.08	1.04
Hamburg	117,817	6	1,830,584	235	204	0.07	5.09	11.1	2.18
Lower Saxony	751,878	15	7,962,775	347	256	0.09	1.99	3.21	1.61
North Rhine-Westphalia	1,813,596	57	17,912,134	1219	1006	0.10	3.14	5.62	1.79
Hesse	664,507	15	6,243,262	569	500	0.11	2.26	8.01	3.54
Rhineland-Palatinate	434,667	14	4,073,679	240	195	0.11	3.22	4.79	1.49
Baden-Württemberg	1,440,334	30	11,023,425	675	491	0.13	2.08	4.45	2.14
Bavaria	2,013,035	61	12,997,204	854	662	0.16	3.03	5.09	1.68
Berlin	441,751	38	3,613,495	420	347	0.12	8.60	9.60	1.12
Brandenburg	257,242	13	2,504,040	159	132	0.10	5.05	5.27	1.04
Saxony	165,932	6	4,081,308	211	181	0.04	3.62	4.43	1.22
Saxony-Anhalt	131,572	5	2,223,081	128	122	0.06	3.80	5.49	1.44
All	8,796,123	275	77,354,808	5194	4214	0.11	3.13	6.71	2.14

The table refers only to the patients from the 12 listed states (column “State”)

^a^ Adjusted notified patients – number of hospitalized TB patients without homeless, prisoners, asylum seeker or refugee status, diagnosed before death and notified in 2017

^b^ per 100,000 people

## Discussion

In order to achieve the goal of assessing the completeness of TB notification in Germany we estimated the level of the underreporting of diagnosed TB in 2013–2017 using two different methods: CRC analysis and comparison of demand for the first-line anti-TB drug PZA based on TB notification data with the recorded consumption of PZA. Further, we compared TB incidence in 2017 estimated based on insurance fund data on in-patient care of TB patients with notified TB incidence in 2017. In summary, the estimated TB underreporting was lowest in the year 2014 (CRC: 3.0%; PZA: 5.3%) and highest in 2016 and 2017 (2016, CRC: 6.0%, PZA: 9.4%; 2017: CRC: 6.6%, PZA: 7.0%), and did not exceed the level of 10% in any of the studied years.

According to WHO, a successful inventory study requires six components: (1) case-based data with reliable personal identifiers, (2) standard case definitions across all care providers, (3) adequate staffing and funding, (4) care providers outside the existing national TB control programs network can be mapped and convinced to participate, (5) expertise in sampling design, data management and data analysis, and (6) at least three fairly independent data sources and sampling of 50% of country areas if CRC is planned.

Our study addressed all of the listed components despite encountered limitations. Due to the availability of only pseudonymous data for this study, we used probabilistic RL on case-based data to comply with component (1). The components 2, 3, and 4, are fulfilled in Germany which is a country with strong public health system where TB is a notifiable disease and case definitions are standardized, there is adequate staffing and funding of the medical system and universal health care. While RKI notification data refers to particular case definition described in the Methods section, in the ARS data the “case” is an isolate with microbiologically confirmed TB which has been addressed by our de-duplication strategy. Component (5) was met as the study team had high expertise in sampling design, data management and data analysis and implemented robust analytical methods. Condition (6) was a source of concern for our study, as unavailability of a third case-based data source as well as incompliance with the assumption of closed population and independence of data sources could have provided results that underestimated the true underreporting estimation. In order to minimize this bias, we (a) used CRC model adjusting for lack of independence between datasets; and (b) developed a “double-pronged” inventory study approach. This approach makes use of all available datasets by not only conducting a CRC analysis, but also seeks to estimate TB reporting completeness via other methods that do not require case-based reporting, and then compares the derived results. Hence, even though there is a possibility that our CRC analysis results could be biased towards lower than real underreporting estimation, the overall result of the “double-pronged” inventory study should present a reliable picture of the underreporting of diagnosed TB in Germany. Our results show that the TB underreporting estimations for 2017 based on the used approaches are consistent with one another and indicate TB underreporting of 7%, which is an increase in comparison to 2013–2014. We observed a discrepancy between TB underreporting estimates based on TB notification data and PZA pharmacy data in the previous years, which was particularly pronounced in 2015 and 2016 (2015: CRC: 4.2%; PZA: − 3.6%; 2016: CRC: 6.0%; PZA: 9.4%). The discrepancies in datasets collected in those years can be associated with demographic changes caused by migration in the years 2014–2016, as immigrants to Germany largely originate from countries with higher TB incidences, and TB notification rates are about 20 times higher among foreign compared with German citizens [34, 35]. Substantial numbers of asylum seekers arrived in Germany in those years and it is possible that the notifications were susceptible to duplications due to frequent transfers of migrants between areas under the jurisdiction of different public health offices. In reference to CRC, the increased immigration causes incompliance with the CRC assumption of the closed population. Therefore, especially for the years 2014–2016 it is important to compare the results of CRC with the results obtained with other methods.

The probabilistic RL of the records from ARS dataset and TB notification dataset indicated an overlap of between 61% (in the years 2013 and 2016) and 65% (2014) between the datasets while every laboratory-confirmed TB case (representing 72.2% of diagnosed TB patients as of 2017) should be notified. It is likely that the missing 35–39% unmatched patients comprise laboratory confirmed patients which have not been notified as well as patients for which the completeness and coherence of the notified and/or ARS data were insufficient to match patients between the registers. This result encourages further attention towards thoroughly reporting of patient characteristics in the future.

The more than doubled number of TB patients in ARS data was observed between the years 2013 and 2017. In those years, the data was collected from the same participating hospitals and medical practices. Potential reasons for such a large increase could include increased testing of vulnerable groups, in particular asylum seekers, and should be further investigated. Smaller but significant increase in the number of TB patients is also visible in the notification data.

We used an independent method based on comparing PZA consumption in Germany to the calculated demand for PZA based on TB notification data in order to verify the reliability of the CRC results. Additionally, we requested aggregated datasets of TB patients from three institutions in Germany (Supplementary Table 1) to compare the TB notification data with reported hospitalized or treated, or insured TB patients. However, three of five acquired aggregated datasets had to be excluded from the study due to unreliable data quality. We are not able to assess whether the registers contain false positive data, and speculate that the patients might have been recorded multiple times due to frequent doctor visits during the disease and/or that primary suspicion of TB might be erroneously reported as the final diagnosis.

Aggregated data has limited use in estimating the disease underreporting level. However, when unique disease case reporting in any other register exceeds those in the TB notification system for a certain population, this indicates underreporting. Insurance data did not imply an overall TB underreporting in Germany, neither in any of the investigated states.

Germany has a population of over 80 million people with a decentralized health system, and very strict data protection laws. This presents challenges for the reporting of TB data, but also for the collection of data from different sources in comparison to countries with smaller population size. The inventory studies from various countries published as of March 2019 involved national TB notification data ([6–8, 13, 14] and others), laboratory data [13], prescription data [13, 18], social insurance data [8, 11] and hospital admission data [6, 7]. In Germany, data acquisition presented a marked challenge. The CRC for the years 2013–2017 was only possible for two TB registers in Germany mostly due to very strict data sharing policies. Since only one request for anonymized, case-based data was met, we conducted CRC violating the method’s assumptions by missing three-source interaction between sources. 3-source CRC studies have been previously conducted in other European and non-European countries and are recommended by WHO. It would be important that appropriate secondary data can be made available for similar public health studies in Germany in the future.

The presented study has certain limitations. First of all, our CRC analysis results have to be interpreted carefully, taking into account that the analysis was performed with only two data sources, and not meeting the assumption of data sources independence, closed population, and perfect RL. The patients from which isolates were collected in ARS data had an increased chance to be present in TB notification data due to legal obligation of laboratories to notify TB patients to the local health authorities. This can lead to underestimation of the TB underreporting and was addressed by choosing CRC model adjusted for lack of independence between data sources. Even though use of inventory studies is expected to result in a more accurate estimation of TB reporting completeness than use of other methods, it is possible to miscalculate TB underreporting level using CRC because of potential errors in TB notification data – e.g. incorrect or duplicated reports. This can lead to both under- and overestimation of the underreporting, depending on the error type. Even though we conducted data de-duplication, fully successful de-duplication can only be assumed in circumstances where the notifications are complete and do not contain errors, which is not the case for TB notification data. In this study, we used three methods to estimate the underreporting of the diagnosed TB patients in Germany (including patients diagnosed post-mortem). However, the study does not address underdiagnosis of TB, which cannot be excluded and could imply that the real TB incidence in Germany is higher than estimated in this study. However, the diagnostic capacity and the use of postmortem diagnosis may reduce the risk of underdiagnosis. The delay between disease onset and diagnosis, which currently cannot be estimated based on the notification data due to incomplete reporting of this information, could contribute to further uncertainty of the presented estimation.

The results of the present TB inventory study for Germany differ from the results of some previously conducted international studies and can likely be attributed to differences in the public health systems of other countries. The TB underreporting rates ranged from 80% in the studies conducted in Greece and 69% in Central Italy to 45% in Egypt, between 20 and 30% in Croatia, Finland, Iraq and Yemen, and less than 20% in China, United Kingdom, Romania, Portugal, and the Netherlands. The lowest estimated TB underreporting to date, of below 4% and below 2%, were reported in inventory studies conducted in Taiwan [11] and Denmark [16], respectively. Despite the differences, multiple study authors have reported similar problems as those observed by us in Germany: dependences between case-based data sources, lack of universal patient identifiers across data sources and, in certain cases, insufficient data quality [14, 36]. Countries where the patients had unique personal identifiers across databases or where the data used was not anonymized presented the highest certainty of the results [8, 11, 16].

Inventory studies conducted in other countries show that the TB underreporting estimated by the WHO differs from the underreporting estimated based on the inventory studies of TB registers (e.g. in the most recent study, published in 2020, TB underreporting in Denmark in 2014–2016 was estimated at 1.3%, in contrast to the WHO estimation of 5.2% for 2016 [8, 37]). The results of the inventory study in Germany also remain in contrast with WHO estimations which indicate TB underreporting of between 11 and 17% in 2013–2017 [1, 37–40]. Potential reasons for this discrepancy include the used methodology: while WHO’s standard adjustments give a rough estimate of disease incidence, these are not based on the actual and up-to-date data sources, as in the case of our inventory study. A further source of discrepancy could be the fact that inventory studies do not account for undiagnosed TB patients. WHO defines > = 90% of TB reporting completeness in countries where TB is a notifiable disease as one of the benchmarks for satisfying TB surveillance system coverage, which according to our study is fulfilled in Germany [41].

## Conclusions

We conclude that more than 90% of estimated TB patients are captured within the German TB surveillance system, and accordingly the TB notification rate is likely a good proxy of the diagnosed TB incidence rate. An increase in underreporting and discrepancies between data sources however should be further investigated and involve additional case-based data sources.

## Supplementary information

**Additional file 1: Supplementary Table 1:** List of the institutions from which case-based data on TB patients in Germany was requested. **Supplementary Method 1** CRC analysis was performed using R package “Rcapture” [23]. Three closed population models (M0, Mt and Mb) assuming no births, deaths, immigration and emigration affecting capture in both data sources within study period were built for each of the reporting years from 2013 to 2017 using “closedp” function with default parameters. Model presenting minimal AIC was chosen to estimate the number of TB patients in Germany. In the years where two models presented identically low AIC, model Mb was chosen because Mt and M0 models implied unrealistically high number of TB patients. Please see the example below for the year 2013: **Supplementary Table 2:** TB patients hospitalized in the years 2013–2017.

## Data Availability

The Data Protection Office of the Robert Koch Institute restricts sharing of any case-based RKI surveillance data externally as the data are only pseudonymized and contain potentially identifying patient information. However, aggregated notification data can be requested from the authors or the Research Data Management Unit of the Robert Koch Institute (MF4@rki.de) under reasonable conditions. The external datasets utilized in the study were made available to the authors in aggregated form by external institutions for the purpose of the study and the authors are not permitted to further share the data.

## Abbreviations

AKI: Akaike’s Information Criterion
ARS: Antibiotic Resistance Surveillance
ATC: anatomical-therapeutic-chemical index
BKK DV: Betriebskrankenkassen Dachverband
CRC: Capture-Recapture
DDD: defined daily dose
MDR-TB: multidrug-resistant tuberculosis
PZA: pyrazinamide
RKI: Robert Koch Institute
RL: record linkage
SHI: statutory health insurance
TB: tuberculosis
WHO: World Health Organization

## References

[1] World Health Organization. Global tuberculosis report 2018 [Internet]. World Health Organization; 2018 [cited 2019 Feb 6]. Available from: https://www.who.int/tb/publications/global_report/en/.

[2] World Health Organization. WHO End TB Strategy [Internet]. World Health Organization. World Health Organization; 2015 [cited 2019 May 13]. Available from: https://www.who.int/tb/post2015_strategy/en/.

[3] Iglesias Gozalo MJ, Rabanaque Hernández MJ, Gómez López LI (2002). Tuberculosis in the Zaragoza province. Estimation by means of the capture-recapture method. Rev Clin Esp.

[4] World Health Organization. Assessing tuberculosis under-reporting through inventory studies. Geneva: World Health Organization [Internet]; 2015. Available from: https://www.who.int/tb/publications/inventory_studies/en/.

[5] Gesetz zur Modernisierung der epidemiologischen Überwachung übertragbarer Krankheiten. In: Bundesgesetzblatt Jahrgang 2017 Teil I Nr 49 [Internet]. 2017 [cited 2019 May 13]. Available from: https://www.bgbl.de/xaver/bgbl/start.xav?start=%2F%2F*%5B%40attr_id%3D%27bgbl117s2615.pdf%27%5D#__bgbl__%2F%2F*%5B%40attr_id%3D%27bgbl117s2615.pdf%27%5D__1557757957459.

[6] Faensen D, Claus H, Benzler J, Ammon A, Pfoch T, Breuer T, et al. SurvNet@RKI - A multistate electronic reporting system for communicable diseases. [cited 2019 May 13]; Available from: https://edoc.rki.de/handle/176904/677. 16645245

[7] World Health Organization. Standards and benchmarks for tuberculosis surveillance and vital registration systems. World Health Organization [Internet]. 2015 [cited 2019 May 13]; Available from: https://www.who.int/tb/publications/standardsandbenchmarks/en/.

[8] Li T, Shewade HD, Soe KT, Rainey JJ, Zhang H, Du X (2019). Under-reporting of diagnosed tuberculosis to the national surveillance system in China: an inventory study in nine counties in 2015. BMJ Open.

[9] Bassili A, Grant AD, El-Mohgazy E, Galal A, Glaziou P, Seita A (2010). Estimating tuberculosis case detection rate in resource-limited countries: a capture-recapture study in Egypt. Int J Tuberc Lung Dis.

[10] Huseynova S, Hashim DS, Tbena MR, Harris R, Bassili A, Abubakar I (2013). Estimating tuberculosis burden and reporting in resource-limited countries: a capture-recapture study in Iraq. Int J Tuberc Lung Dis.

[11] Lo H-Y, Yang S-L, Chou P, Chuang J-H, Chiang C-Y (2011). Completeness and timeliness of tuberculosis notification in Taiwan. BMC Public Health.

[12] Bassili A, Al-Hammadi A, Al-Absi A, Glaziou P, Seita A, Abubakar I (2013). Estimating the tuberculosis burden in resource-limited countries: a capture-recapture study in Yemen. Int J Tuberc Lung Dis.

[13] Cojocaru C, van Hest NA, Mihaescu T, Davies PD (2009). Completeness of notification of adult tuberculosis in Iasi County, Romania: a capture-recapture analysis. Int J Tuberc Lung Dis.

[14] van Hest NAH, Smit F, Baars HWM, De Vries G, De Haas PEW, Westenend PJ (2007). Completeness of notification of tuberculosis in The Netherlands: how reliable is record-linkage and capture–recapture analysis?. Epidemiol Infect.

[15] Melosini L, Vetrano U, Dente FL, Cristofano M, Giraldi M, Gabbrielli L (2012). Evaluation of underreporting tuberculosis in Central Italy by means of record linkage. BMC Public Health.

[16] Straetemans M, Bakker MI, Alba S, Mergenthaler C, Rood E, Andersen PH, et al. Completeness of tuberculosis (TB) notification: Inventory studies and capture-recapture analyses, six European Union countries, 2014 to 2016 [Internet]. Vol. 25, Eurosurveillance. European Centre for Disease Prevention and Control (ECDC); 2020 [cited 2020 Aug 26]. p. 1. Available from: https://www.eurosurveillance.org/content/10.2807/1560-7917.ES.2020.25.12.1900568. 10.2807/1560-7917.ES.2020.25.12.1900568 PMC7118341 32234122

[17] Van Hest NAH, Story A, Grant AD, Antoine D, Crofts JP, Watson JM (2008). Record-linkage and capture-recapture analysis to estimate the incidence and completeness of reporting of tuberculosis in England 1999–2002. Epidemiol Infect.

[18] Lytras T, Spala G, Bonovas S, Panagiotopoulos T (2012). Evaluation of Tuberculosis Underreporting in Greece through Comparison with Anti-Tuberculosis Drug Consumption. PLoS One.

[19] Capture-recapture and Removal Methods for Sampling Closed Populations - Gary C. White - Google Books [Internet]. [cited 2020 Aug 26]. Available from: https://books.google.de/books?hl=en&lr=&id=dHPwAAAAMAAJ&oi=fnd&pg=PR9&dq=capture+recapture+method&ots=KurJwDMVeP&sig=IoW82Ex2NboJQkUirhw2Gcxs400#v=onepage&q&f=false.

[20] Chao A, Tsay PK, Lin SH, Shau WY, Chao DY (2001). The applications of capture-recapture models to epidemiological data. Stat Med.

[21] World Health Organization. WHO Collaborating Centre for Drug Statistics Methodology [Internet]. [cited 2019 Jul 4]. Available from: https://www.whocc.no/atc_ddd_index/.

[22] Schmidt D, Kollan C, Stoll M, Stellbrink H-J, Plettenberg A, Fätkenheuer G (2015). From pills to patients: an evaluation of data sources to determine the number of people living with HIV who are receiving antiretroviral therapy in Germany. BMC Public Health.

[23] Baillargeon S, Rivest L-P (2007). The Rcapture Package: Loglinear Models for Capture-Recapture in R. J Stat Softw.

[24] World Health Organization. Introduction to drug utilization research [Internet]. World Health Organization; 2003 [cited 2019 Apr 8]. Available from: http://apps.who.int/medicinedocs/en/d/Js4876e/.

[25] Brodhun B, Altmann D, Hauer B, Haas W. Bericht zur Epidemiologie der Tuberkulose in Deutschland für 2017 [Internet]. Berlin; 2018. Available from: https://www.rki.de/DE/Content/InfAZ/T/Tuberkulose/Download/TB2017.pdf?__blob=publicationFile.

[26] Noll I, Schweickert B, Abu Sin M, Feig M, Claus H, Eckmanns T (2012). Daten zur Antibiotikaresistenzlage in Deutschland. Bundesgesundheitsblatt - Gesundheitsforsch - Gesundheitsschutz.

[27] R Core Team R. R: A Language and Environment for Statistical Computing [Internet]. Team RDC, editor. R Foundation for Statistical Computing. Vienna, Austria: R Foundation for Statistical Computing; 2018. 409. (R Foundation for Statistical Computing; vol. 1). Available from: http://www.r-project.org.

[28] Borg A, Sariyar M (2010). The RecordLinkage package: detecting errors in data. R J.

[29] Schaberg T, Bauer T, Castell S, Dalhoff K, Detjen A, Diel R (2012). Empfehlungen zur Therapie, Chemoprävention und Chemoprophylaxe der Tuberkulose im Erwachsenen- und Kindesalter. Pneumologie.

[30] Canty A, Ripley B (2020). boot: Bootstrap R (S-Plus) Functions.

[31] Davison A, Hinkley D (1997). Bootstrap Methods and Their Applications.

[32] Statistisches Bundesamt. 2019. https://www.destatis.de/EN/Home/_node.html.

[33] Rao S (2009). Tuberculosis and patient gender: An analysis and its implications in tuberculosis control. Lung India.

[34] Brodhun B, Altmann D, Hauer B, Fiebig L, Haas W. Bericht zur Epidemiologie der Tuberkulose in Deutschland für 2015 [Internet]. Berlin; 2016. Available from: https://www.rki.de/DE/Content/InfAZ/T/Tuberkulose/Download/TB2015.pdf?__blob=publicationFile. 10.1055/s-0034-1391922 25970119

[35] Fiebig L, Hauer B, Brodhun B, Altmann D, Haas W (2016). Tuberculosis in Germany: A declining trend coming to an end?. Eur Respir J.

[36] Van Hest NAH, Story A, Grant AD, Antoine D, Crofts JP, Watson JM (2008). Record-linkage and capture-recapture analysis to estimate the incidence and completeness of reporting of tuberculosis in England 1999-2002. Epidemiol Infect.

[37] World Health Organization. Global tuberculosis report 2017 [Internet]. World Health Organization. 2017 [cited 2018 Jun 7]. Available from: http://www.who.int/tb/publications/global_report/en/.

[38] World Health Organization. Global Tuberculosis Report 2015, 20th ed. [Internet]. 2015. Available from: http://www.who.int/iris/handle/10665/191102.

[39] World Health Organization. Global Tuberculosis Report 2016 [Internet]. 2016 [cited 2019 May 15]. Available from: https://apps.who.int/medicinedocs/en/d/Js23098en/.

[40] World Health Organization. Global Tuberculosis Report 2014 [Internet]. 2014. Available from: http://www.who.int/iris/handle/10665/137094.

[41] World Health Organization. Standards and benchmarks for tuberculosis surveillance and vital registration systems. Geneva: World Health Organization [Internet]. 2015. Available from: https://www.who.int/tb/publications/standardsandbenchmarks/en/.

