# Supplementary material for: Completeness of tuberculosis case notifications in Germany in 2013–2017: first results of an inventory study
Source: BMC Infect Dis. 2020 Oct 17;20:766. doi: 10.1186/s12879-020-05467-9 (PMC7568379; doi:10.1186/s12879-020-05467-9)
Supplement: Supplementary file 1 — Additional file 1: Supplementary Table 1: List of the institutions from which case-based data on TB patients in Germany was requested. Supplementary Method 1 CRC analysis was performed using R package “Rcapture” [23]. Three closed population models (M0, Mt and Mb) assuming no births, deaths, immigration and emigration affecting capture in both data sources within study period were built for each of the reporting years from 2013 to 2017 using “closedp” function with default parameters. Model presenting minimal AIC was chosen to estimate the number of TB patients in Germany. In the years where two models presented identically low AIC, model Mb was chosen because Mt and M0 models implied unrealistically high number of TB patients. Please see the example below for the year 2013: Supplementary Table 2: TB patients hospitalized in the years 2013–2017. [file 12879_2020_5467_MOESM1_ESM.docx]

Supplement

**Supplementary Table 1:** List of the institutions from which case-based data on TB patients in Germany was requested.

| Number | Type of institution | Acquired datasets | Reason of lack of dataset acquisition |
| --- | --- | --- | --- |
| 1 | SHI company | - | rejected the data-sharing request due to lack of capacity |
| 2 | SHI company | - | Stopped responding to e-mails or calls without data being sent |
| 3 | Health insurance fund | 3 aggregated datasets (no case-based data due to internal data-sharing policies) |  |
| 3 | Statistical agency | - | fee and a waiting time that exceeded the funding period of the study |
| 4 | Statistical agency | aggregated dataset (no case-based data due to internal data-sharing policies) |  |
| 5 | Research institute for medical care | aggregated dataset (no case-based data due to internal data-sharing policies) |  |
| 7 | Unit of RKI | Acquired case-based dataset |  |
| 8 | Company collecting pharmacy data | Aggregated dataset available at RKI |  |

**Supplementary Method 1**

CRC analysis was performed using R package “Rcapture” (28). Three closed population models (M0, Mt and Mb) assuming no births, deaths, immigration and emigration affecting capture in both data sources within study period were built for each of the reporting years from 2013 to 2017 using “closedp” function with default parameters. Model presenting minimal AIC was chosen to estimate the number of TB patients in Germany. In the years where two models presented identically low AIC, model Mb was chosen because Mt and M0 models implied unrealistically high number of TB patients. Please see the example below for the year 2013:

| Model | Abundance | Standard deviation | Deviance | Degrees of freedom | AIC |
| --- | --- | --- | --- | --- | --- |
| M0 | 18962.0 | 939.3 | 4.3E+03 | 1 | 4322.8 |
| Mt | 7051.7 | 239.1 | -2.6E-13 | 0 | 30.8 |
| Mb | 4548.0 | 3.3 | 1.7E-13 | 0 | 30.8 |

**Supplementary Table 2:** TB patients hospitalized in the years 2013-2017.

| Year | Number of hospitalized TB patients in Germany | Total number of TB patients in Germany | Percentage of hospitalized TB patients in Germany |
| --- | --- | --- | --- |
| 2013 | 3668 | 4345 | 84% |
| 2014 | 3914 | 4529 | 86% |
| 2015 | 5195 | 5837 | 72% |
| 2016 | 5346 | 5926 | 90% |
| 2017 | 4960 | 5495 | 81% |
